# Pattern of HER2 and HER3 Overexpression in Patients with Pancreatic Ductal Adenocarcinoma

**DOI:** 10.3390/medicina62020251

**Published:** 2026-01-24

**Authors:** Ioan Cătălin Bodea, Andra Ciocan, Florin Vasile Zaharie, Raluca Bodea, Ștefan Ursu, Răzvan Alexandru Ciocan, Răzvan George Bogdan, Alin Fetti, Sorana D. Bolboacă, Filip Cristian Tocoian, Bobe Petrushev, Ana Maria Fit, Ioana Rusu, Roxana Liana Popa, Nadim Al Hajjar

**Affiliations:** 1 Department of Surgery, “Iuliu Hațieganu” University of Medicine and Pharmacy, 400162 Cluj-Napoca, Romania; BODEA_IOAN_CATALIN@elearn.umfcluj.ro (I.C.B.); zaharie.vasile@umfcluj.ro (F.V.Z.); ursu_stefan@elearn.umfcluj.ro (Ș.U.); fetti.alin@umfcluj.ro (A.F.); nadim.alhajjar@umfcluj.ro (N.A.H.); 2 Department of Surgery, “Octavian Fodor” Regional Institute of Gastroenterology and Hepatology, 400162 Cluj-Napoca, RomaniaTOCOIAN_FILIP_CRISTIAN@elearn.umfcluj.ro (F.C.T.); 3 Department of Surgery-Practical Abilities, “Iuliu Hațieganu” University of Medicine and Pharmacy, 400337 Cluj-Napoca, Romania; Razvan.Ciocan@umfcluj.ro; 4 Plastic Surgery Department, “Victor Babes” University of Medicine and Pharmacy, 300041 Timisoara, Romania; 5 County Clinical Emergency Hospital Pius Branzeu, 300723 Timisoara, Romania; 6 Department of Medical Informatics and Biostatistics, “Iuliu Hatieganu” University of Medicine and Pharmacy Cluj-Napoca, 400349 Cluj-Napoca, Romania; sbolboaca@umfcluj.ro; 7 Department of Pathology, “Octavian Fodor” Regional Institute of Gastroenterology and Hepatology, 400162 Cluj-Napoca, Romania

**Keywords:** HER2, HER3, pancreatic ductal adenocarcinoma (PDAC), molecular pathway, membrane receptors, targeted therapy

## Abstract

*Background and Objectives*: Pancreatic ductal adenocarcinoma (PDAC) is among the most aggressive, heterogeneous, and lethal human malignancies, underscoring the urgent need for novel, targeted therapeutic strategies for neo(adjuvant) individualized treatment. The epidermal growth factor receptor family (ErbB) is directly involved in abnormal cell proliferation and tumor growth. The overexpression and amplification of HER2 and HER3 have emerged as key molecular events in pancreatic ductal adenocarcinoma. The aim of this study was to evaluate these membrane receptors’ overexpression in relation to pTNM staging, perineural and lymphovascular invasion, and tumor volume in order to obtain the immunohistochemical profile and enhance the development of a targeted and personalized therapy. *Materials and Methods*: An observational analytical cohort study included patients with histopathologically naïve, confirmed PDAC who underwent cephalic pancreatoduodenectomy at a national high-volume referral center between 2017 and 2022. Archived surgical specimens were retrieved and examined using a tissue microarray technique in two separate pathology departments by two independent pathologists. *Results*: HER2 positivity was found in 25 cases, of which 84% had lymphatic invasion, 50% had vascular invasion, and 84% had perineural invasion. Patients with HER3 positivity had lymphatic invasion (82.5%), perineural invasion (79.4%), and vascular invasion (38.1%). Combined HER2 and HER3 positivity was present in 19 cases, and these patients had 84.2% perineural invasion. *Conclusions*: HER2 and HER3 overexpression often coexisted with pathological features, such as perineural invasion, in cases of combined HER2 and HER3 positivity. These findings support the involvement of the ErbB receptor family in pancreatic carcinogenesis and suggest their potential as targets for future (neo)adjuvant therapeutic strategies.

## Simple Summary

The new era of targeted treatment is continuously developing to include protein receptors in the standard protocols before and after surgical treatment in the endeavor of finding a cure for pancreatic cancer. Pancreatic cancer presents high heterogeneity and mortality rates. Epidermal growth factor family receptors (ErbBs) represent a common carcinogenic pathway, in particular, HER2 and HER3. Their overexpression increases tumor burden. This molecular profiling enhances the development of new-generation therapies dependent on ErbBs.

## 1. Introduction

Pancreatic cancer (PC), or pancreatic ductal adenocarcinoma (PDAC), is the sixth leading cause of cancer-related deaths worldwide [1]. In 2025, it represented 3.3% of global cancer cases, with about 510,922 new diagnoses and 467,409 deaths [1]. The one-year overall survival (OS) rate for individuals with PDAC is around 24%, but the five-year OS rate diminishes to approximately 13% [1]. Western Europe has a higher incidence age-standardized rate compared to Northern America, with a rate of 10.1 per 100,000 people in males and 7.7 per 100,000 people in females compared with 9.6 per 100,000 individuals in males and 7.4 per 100,000 individuals in females [2]. The etiology of cephalopancreatic PDAC remains insufficiently elucidated due to multiple contributing factors; however, several risk factors have been identified, including diabetes mellitus, ethnicity, tobacco consumption, age, dietary elements, alcohol consumption, metabolic factors such as obesity, familial history, genetic predispositions, Helicobacter pylori infection, non-O blood group, and chronic pancreatitis [3,4].

Surgery remains the principal curative method for ductal adenocarcinoma of the head of the pancreas; however, only 15–20% of tumors are resectable at the time of the diagnosis, and the prognosis remains poor even for R0 resections [5,6,7]. Adjuvant chemotherapy has limited advantages, resulting in a median survival time of 12 months. Conventional regimens such as mFOLFIRINOX followed by capecitabine have shown limited positive effects, with poor effects on decreasing tumor invasion in borderline tumors [8,9]. Consequently, it is essential to implement targeted treatments to improve overall survival and disease-free survival.

Several studies have examined the essential elements of protein receptors implicated in the aberrant proliferation of malignant tissue. Growth factors are crucial for cell-to-cell communication via interactions with cell surface receptors, sustaining embryonic tissue induction, prolonging the life of the cell mass, controlling apoptosis, enabling tissue differentiation, and guiding cell migration and proliferation. The epidermal growth factor family consists of four members: EGFR (ErbB1, HER1), ErbB2 (HER2), ErbB3 (HER3), and ErbB4 (HER4). ErbB2 seems to be the preferred dimerization partner for other members of the EGFR family [6,7].

HER2 is a proto-oncogene located on chromosome 17q21 that encodes ERBB2, a member of the epidermal growth factor receptor (EGFR/ERBB) family of transmembrane receptors with intrinsic tyrosine kinase activity. Unlike other ERBB receptors, HER2 has no known ligand and functions as a preferred dimerization partner, amplifying downstream signaling through pathways such as PI3K/AKT and MAPK/ERK. Overexpression or amplification of HER2 promotes uncontrolled cellular proliferation, survival, and metastatic potential, and it plays a well-established oncogenic role in several epithelial malignancies [10].

Amplification occurs in 15–20% of invasive breast cancers and is significantly correlated with HER2 protein overexpression [10,11]. HER2 amplification is associated with an unfavorable prognosis, correlating with higher rates of recurrence and death, and serves as a predictive biomarker for responsiveness to specific chemotherapeutic regimens [9,10,11,12]. Current diagnostic and therapeutic recommendations include HER2 evaluation in stomach and colorectal malignancies; however, its assessment in periampullary tumors, implying pancreatic ductal adenocarcinoma, ampulla of Vater adenocarcinoma and distal cholangiocarcinoma, has not yet been thoroughly investigated. The incidence of HER2 positivity in cholangiocarcinoma has been reported to range between 5% and 20% [13,14,15]. It is the only prognostic indicator for the therapeutic advantages of certain targeted medicines, including trastuzumab, lapatinib, and pertuzumab. The importance of HER2 in gastric tumors for prognostic and therapeutic applications has produced inconsistent results [16,17,18,19,20]. The HER2 pathway is one of the most neglected and underdiagnosed carcinogenic pathways in pancreatic cancer. HER2 lacks particular ligands; hence, it can only form heterodimers when activated by other receptors, such as EGFR, HER3, or HER4 [21,22].

HER3 is unique within this family as it is the only member devoid of catalytic kinase activity [23]. Significant evidence, mainly from experimental models, now suggests that its non-catalytic actions are crucial in several cancers associated with its HER family counterparts [24]. Failure to satisfy the standard requirements for an oncogene has allowed HER3’s tumor-promoting actions to circumvent oncological therapies [25]. Upon dimerization with HER2, HER3 promotes an allosteric interaction that activates the HER2 kinase domain, hence commencing intracellular signaling [26]. HER3 is vital for HER2-mediated transformation, and its presence is critical for the continued development and proliferation of cancer cells in HER2-overexpressing tumors. In two separate knockdown scenarios, the absence of functional HER3 in HER2-amplified breast cancer cell lines resulted in a reversion of transformed growth similar to that observed with the loss of HER2 [25,27]. In contrast, the attenuation of EGFR expression by shRNA silencing did not affect HER2-driven tumors, indicating that HER3 is essential for HER2-mediated carcinogenesis [25]. Consequently, the majority of HER2-amplified breast carcinomas coexpress HER3; however, EGFR coexpression is inconsistent [27,28].

Worldwide, research has sought to identify the roles of HER2 and HER3 receptors in abnormal cell proliferation in pancreatic cancer, to introduce targeted anti-HER2 and anti-HER3 therapeutic regimens into neoadjuvant or adjuvant treatment protocols. The current literature on HER2 and HER3 expression in PDAC is limited, with no reports on the Romanian PDAC cohort. Our study aimed to evaluate both the presence and overexpression of HER2 and HER3 in patients diagnosed with cephalopancreatic ductal adenocarcinoma who did not previously receive systemic treatment, were naïve at the time of surgery, and underwent either the pylorus preserving Whipple procedure or the classic Whipple procedure, to establish the roles of these protein receptors in cancer cell proliferation and differentiation in conjunction with patient characteristics.

## 2. Materials and Methods

### 2.1. Study Design and Cohort

An observational analytical cohort study was conducted on naïve patients with a histopathological diagnosis of pancreatic ductal adenocarcinoma who were referred to the Regional Institute of Gastroenterology and Hepatology “Octavian Fodor” (IRGH) Cluj-Napoca over a period of 5 years, from 2017 until 2022, for specific surgical treatment. All patients, regardless of age and sex, underwent cephalic pancreatoduodenectomy (the Whipple procedure), and their resection specimens were stored in the institute’s pathology department. There, they were further evaluated for study eligibility. IRGH Cluj-Napoca is a high-volume center for hepatobiliopancreatic surgery in Romania and a referral center for pancreatic cancer.

The Whipple procedure has several indications besides pancreatic ductal adenocarcinoma, such as distal cholangiocarcinoma, duodenal adenocarcinoma, ampulla of Vater adenocarcinoma, neuroendocrine tumors, acute necrotic hemorrhagic pancreatitis, pseudotumoral pancreatitis, and other rare conditions. From the eligible cohort, 106 PDAC cases that met the inclusion criteria were included in the final analysis. Selection was based on histological quality, and only cases with well-preserved, representative tumor tissue identified on hematoxylin–eosin–stained sections were further analyzed. The inclusion criteria were as follows: positive histopathological diagnosis of pancreatic ductal adenocarcinoma on endoscopic ultrasound biopsy prior to surgery, presence of per primam resectable tumors, no prior treatment with neoadjuvant chemotherapy or immunotherapy, and pancreatoduodenectomy with curative intent. Exclusion criteria consisted of incomplete medical history; surgical procedures other than the Whipple procedure, performed with palliative intent; treatment with neoadjuvant chemotherapy (capecitabine and FOLFOX/FOLFIRINOX) or any immunotherapy performed in clinical trials in phases I, II, or III; and locoregional vascular invasion in the superior mesenteric artery or vein of more than 180 degrees.

Medical records were used to collect demographic data (age and sex); histopathological details from the surgically resected specimens after the Whipple procedure; tumor differentiation grades (G1—well differentiated (low grade), G2—moderately differentiated (intermediate grade), G3—poorly differentiated (high grade), G4—undifferentiated (high grade)); tumor diameters (DCC—craniocaudal diameter, DAP—anteroposterior diameter, DLL—lateral diameter); pTNM staging (tumor, nodes, distant metastases); lymphatic vessels invasion (L0—without or L1—microscopic invasion); vascular invasion (other than the mesenteric axis; V0—without or V1—tumor emboli at the vascular level); perineural invasion (Pn0—without or Pn1—microscopic invasion at the level of the nerve sheath/nervous structures included in the resection specimen); resection margins (R0—free of tumor tissue or R1—microscopic cells present on the transection margins); number of days of hospitalization; whether death occurred during the hospitalization period, etc.

### 2.2. HER2 and HER3 Evaluation

Every tumor resection specimen from every patient operated on, along with the related immunohistochemistry microscopy plates, are stored at the institute’s pathology laboratory for ten years after surgery. For each patient, 10 to 29 plates containing tumoral mass slices together with lymph nodes, healthy pancreatic tissue, and tumoral tissue samples were prepared. The plates included in the study were independently analyzed by two separate pathologists from the Pathology Department, who then decided by consensus on only one plate out of two samples to be forwarded for further investigation. Along with the evaluated plate, the corresponding paraffin blocks were selected to obtain tumor samples. For each plate, the area with the highest tissue density was marked to obtain the best tumor tissue sample. The tissue was removed from paraffin blocks using a 2 mm puncher and inserted into a new paraffin block with 24 spaces corresponding to the 24 cases on each block. The first case in each block was a standard control, yielding 23 cases per paraffin block. The obtained blocks were incubated at 56–58 °C for 10–15 min to anneal paraffin. After sliding the first plate from the block, HER2 and HER3 antibody solutions were applied to 23 cases per plate (a total of 5 plates), which resulted from the sectioning of the paraffin blocks. The microarray technique (TMA) was used to assess HER2 or HER3 overexpression. The plates were examined with Ventana BenchMark Ultra for HER2 using anti-HER2/neu rabbit monoclonal primary antibody (ref:760-4390, lot: V0004122, sn:294680—ROCHE) and with BondMax for HER3 using HER3 anti-c-erbB3 rabbit monoclonal antibody (clone SP71, SIGMA SAB5500081-100UL) in the institutional laboratory. For the second look, the plates were further examined with the same antibody solution kit for the evaluation of HER2 and HER3 overexpression in a second pathology department of a distinct institution using Ventana BechMark Ultra. LeicaOptics provided the microscopic kit used for all determinations. After collecting all data, the cases were examined twice to obtain a certain morphophonological diagnosis.

In the absence of a validated and universally accepted HER2 or HER3 immunohistochemical scoring system for pancreatic ductal adenocarcinoma, HER2 and HER3 expression was evaluated using a semiquantitative membrane expression scoring approach adapted to the specific histopathological characteristics of PDAC. Each plate was classified as having 0 (non-existent), 1+ (low), 2+ (high), or 3+ (ultra-high) positivity in each laboratory, following the same protocol for HER2 and HER3. HER2 and HER3 positivity was reported using the lowest value in the case of a borderline result (e.g., between 1+ and 2+ was reported as 1+, and between 2+ and 3+ was reported as 2+) to obtain a certain diagnosis. The pathologists who read the plates had similar experience and expertise and were aware that the patients had pancreatic ductal adenocarcinoma but were blinded to the patients’ medical records.

### 2.3. Statistical Analysis

Data were reported according to their type: qualitative data as counts and percentages and quantitative data as either mean ± standard deviation or median with interquartile range [Q1–Q3], depending on their distribution (assessed using the Shapiro–Wilk test). Tumor volume was estimated by assuming an elliptical shape using the following formula: V = (4/3) × PI× (DCC/2) × (DAP/2) × (DLL/2), where DCC = craniocaudal diameter, DAP = anteroposterior diameter, and DLL = laterolateral diameter.

An exploratory analysis was conducted, and the following were compared: men and women, given the hypothesis that sex characteristics may differ; patients with positive or negative HER2 and patients with positive or negative HER3. Negative HER2 or HER3 was defined as class 0; otherwise, the patients were considered positive. In our analysis, positivity was defined as any result ≥ 1+, to capture receptor expression rather than imply therapeutic eligibility. This strategy reflects real-world diagnostic challenges in PDAC, particularly given limited tissue availability and intratumoral heterogeneity. Pancreatic tumor sampling, especially via endoscopic ultrasound-guided fine-needle biopsy, is associated with a significant risk of non-representative tissue acquisition. In this context, we consider the inclusion of 1+ cases as biologically and diagnostically relevant, supporting more comprehensive membrane receptor profiling that may improve histopathological characterization and inform subsequent diagnostic or therapeutic decision-making.

The comparisons of the HER2 and HER3 groups were conducted assuming that the characteristics may differ between groups. Group comparisons were performed using the Student’s *t*-test for independent samples when quantitative data followed a normal distribution, the Mann–Whitney U test when quantitative data deviated from normality, and the Chi-squared test or Fisher’s exact test for categorical data, depending on the expected cell frequencies. All analyses were performed using Statistica software (version 13, StatSoft, Tulsa, OK, USA), with two-tailed tests and a significance threshold of 5% (*p*-value < 0.05).

## 3. Results

A total of 106 patients aged 36–83 years were evaluated. Most tumors were G2 and T2. No statistically significant differences were observed between males and females, except for metastasis, which was observed exclusively in 3 women (Table 1). None of the patients in the investigated cohort had undifferentiated G4 tumors. The tumor burden was similar between the two sexes, with a median of 6.5 cm in males and 6.8 cm in females. Advanced tumor stages were encountered with T2 in over half of the subjects in both groups, and T3 in over a quarter of the subjects in both groups. No statistically significant difference was observed between males and females regarding N1 (45.8% in males vs. 53.2% in females) and N2 (37.3% in males vs. 31.9% in females). A total of 9 patients died from cancer-related causes directly (6 males, 3 females).

Approximately three-quarters of patients had poor HER2 positivity, with HER2 1+ and 2+ (11 patients, 10.4%), and only three patients had high expression (HER2 3+, 2.8%). The patients with and without HER2 positivity had similar demographic and clinical characteristics (Table 2). Lymphovascular invasion (L1) was observed in 84 HER2-positive patients and 76% of HER2-negative patients. Vascular invasion (V1) was present in more HER2-positive specimens compared with negative ones (52% to 42%), and perineural invasion (Pn1) was emphasized in 84% of HER2-positive cases. Without exception, patients with positive HER2 survived during hospitalization.

**Figure 1:**
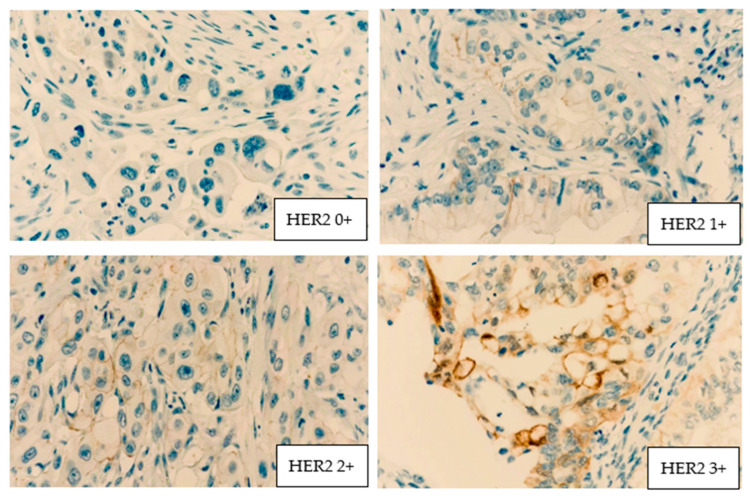
HER2 positivity grades. HER2 0+ = no staining or incomplete and barely perceptible membrane staining in <10% of tumor cells. HER2 1+ = incomplete and scarcely perceptible membrane staining in >10% of tumor cells. HER2 2+ = weak–moderate complete membrane staining in >10% of tumor cells or intense staining in <10% of tumor cells. HER2 3+ = complete and intense membrane staining in >10% of tumor cells.

Four examples of microscopy plates with HER2 positivity are presented in Figure 1.

The overexpression of HER3 was more frequently observed in the investigated cohort, with a similar percentage of patients with HER3 2+ (24, 22.6%) or 3+ (22, 20.8%), and less frequently as HER3 1+ (17, 16%) and no overexpression in 43 patients (40.6%). Lymphovascular invasion (L1) was identified in 82% for the HER3-positive vs. 72% for the HER3-negative group, similar to perineural invasion (79.4% HER3-positive; 65% HER3-negative), with significant N1 positivity for HER3-positive cases in 52% vs. 44%, demonstrating a tendency toward early locoregional invasion. Patients with and without HER3 positivity had similar demographic and clinical characteristics (Table 3).

**Figure 2:**
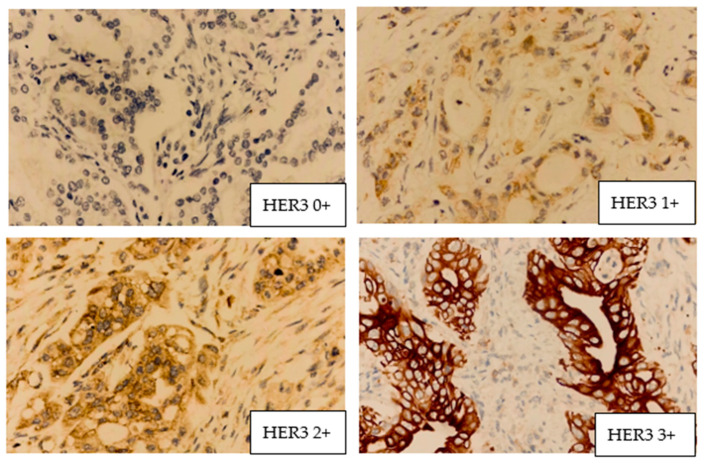
HER3 positivity grades. HER2 0+ = no staining or incomplete and barely perceptible membrane staining in < 10% of tumor cells. HER2 1+ = incomplete and barely perceptible membrane staining in >10% of tumor cells. HER2 2+ = weak–moderate complete membrane staining in >10% of tumor cells or intense staining in <10% of tumor cells. HER2 3+ = complete and intense membrane staining in >10% of tumor cells.

Four examples of microscopy plates with HER3 positivity are presented in Figure 2.

Cases with combined HER2 and HER3 positivity (n = 19) had a total of over 80% perineural invasion of the entire number of specimens evaluated. Combined HER2 and HER3 positivity cases showed advanced T stage (36.8% T3) and N stage (N1 in 63.2% vs. 46%) but showed similar tumor volume 6.3 vs. 6.6 cm^3^ without statistically significant difference (*p* = 0.866) (Table 4).

## 4. Discussion

Among patients with cephalopancreatic ductal adenocarcinoma, similar patterns were observed in male and female patients, with no sex-related differences in the distribution of HER2 or HER3 positivity.

The absence of tumor cells in nearby lymph nodes was observed in 16% of our cohort, consistent with the 17% observed in previous studies [29,30]. N1+ or N2 cases were present in 84%, a frequency higher than in the reported literature [31,32,33]. In Takahashi’s study, 89.1% of cases presented with perineural invasion (Pn1) [34,35], whereas in our study, it was observed in 73.6% of cases (Table 1). Regarding lymphatic invasion (L1), the rate of L1 was 78.2%, and the rate of vascular invasion (V1) was 44.3% (Table 1), consistent with the literature, which reported rates of about 73% for L1 and 49% for V1 [30,36,37]. For HER2-positive cases, the main differentiation grade was G2, the median age was approximately 63 years, the T stage was T2 and T3, and the N stage was N1 for lymph nodes assessment. A considerable number of cases (21/25) were L1, vascular invasion (V1) was positive in half the cases (52%), and perineural invasion (Pn1) was observed in 84% of HER2-positive cases (Table 2). In patients with HER3 overexpression, G2 was the main differentiation grade, while T2-T3 was the main grade for local tumor invasion and N1 was the main grade for positive lymph nodes. In the same group, lymphatic invasion was observed in the majority of cases (52/63 cases with L1), 24 cases (38.1%) were V1, and 50 cases had perineural invasion (79.4%) (Table 3). In the entire cohort, 19 patients had both HER2 and HER3 overexpression. In these cases, the main differentiation grade was G2; the T stage ewas T2 or T3 the N stage was N1 for lymph nodes; 16/19 cases were L1; in half of the cases, V1 was found; and 16 cases were Pn1.

Despite notable progress in the early detection of solid malignancies over the past three decades, pancreatic cancer remains a major challenge for effective oncological management. By 2030, it is projected to become the second leading cause of cancer-related mortality, surpassed only by lung cancer [38]. Owing to its heterogeneous biological behavior, retroperitoneal location, nonspecific clinical presentation, and the absence of reliable screening strategies, most pancreatic tumors localized in the head are diagnosed at advanced, often unresectable stages. Consequently, overall survival remains poor, with 5-year survival rates at approximately 10%, which is largely attributable to delayed diagnosis and the limited efficacy of current therapeutic modalities [30].

Approximately 60–70% of pancreatic ductal adenocarcinomas are localized in the head of the pancreas or the uncinate process. This underscores the need for optimized neoadjuvant treatment algorithms to downstage tumors and cytoreduction, potentially guided by inhibition or modulation of overexpressed molecular targets. To date, only a limited number of HER family inhibitors, primarily EGFR-directed tyrosine kinase inhibitors (TKIs) such as erlotinib, lapatinib, and gefitinib, have received FDA approval (Food and Drug Administration, the U.S. federal agency) for use in combination with gemcitabine-based chemotherapy in patients with locally advanced, unresectable, or metastatic pancreatic cancer. Erlotinib and gefitinib exhibit the strongest activity against EGFR, whereas lapatinib demonstrates dual inhibitory activity against both EGFR and HER2 [30,34].

Given the therapeutic success of HER2- and HER3-targeted inhibitors in breast and gastric cancers and the documented overexpression of multiple ERBB receptors in pancreatic ductal adenocarcinoma, ERBB-family blockade represents a biologically plausible and potentially valuable therapeutic approach in this malignancy. Moreover, additional knowledge of ERBB is essential to enhance the antitumor response [30,31]. Consequently, acquiring a thorough immunohistochemical profile for each patient is crucial for determining targeted treatment based on the overexpression or amplification of membrane receptors. Our data indicate no statistically significant association between pTNM staging and HER2 or HER3 overexpression. The distribution of T stages was dominated by T2 and T3 tumors, regardless of HER2 or HER3 positivity. Lymph node involvement was most frequently classified as N1, showing similar patterns across HER2/HER3 expression groups (Table 2 and Table 3) and between sexes (Table 1). The presence of lymphatic invasion and vascular invasion in our cohort indicates advanced stages. Perineural invasion was observed in 78 cases (73.6%), suggesting a higher overall risk of recurrence.

In 2024, the FDA announced the approval of the innovative HER2/HER3 bispecific antibody, zenocutuzumab (bizengri), for the treatment of adults with advanced, unresectable, or metastatic non-small cell lung cancer (NSCLC) or pancreatic ductal adenocarcinoma (PDAC) possessing a neuregulin 1 (NRG1) gene fusion that had progressed after previous systemic therapy [35]. Enocutuzumab is a bispecific monoclonal antibody that targets the extracellular domains of HER2 and HER3, thereby preventing the formation of HER2/HER3 heterodimers and blocking NRG1 binding to HER3. This method suppresses cellular growth and the PI3K-AKT-mTOR signaling pathway. Univariate analysis revealed that the median survival time for individuals with HER3 overexpression was 37.2 months, compared to 58.6 months for those with HER3-negative samples (*p*-value = 0.008). HER3 overexpression, lymph node metastases, and increased serum CA19-9 levels were identified as independent prognostic indicators associated with worse outcomes in multivariate survival analysis [35,36]. In our study, HER3 positivity was identified in 63 patients, representing roughly 60% (Table 1).

The strengths of this study include a well-defined, systemic treatment-naïve surgical cohort with histopathologically confirmed cephalopancreatic ductal adenocarcinoma treated uniformly with curative-intent pancreatoduodenectomy (the Whipple procedure) in a Romanian national high-volume referral center. Strict inclusion criteria and selection based on histological quality ensured a homogeneous cohort and reliable pathological and immunohistochemical analyses. Our study refines and expands the existing knowledge through a standardized tissue microarray–based approach applied to a standardized, well-characterized Romanian surgical cohort. Despite its rigorous design, our study had some limitations that warrant acknowledgment. An important limitation of our study is its retrospective design, based on routinely collected healthcare data. As a result, the availability, completeness, and standardization of clinical and follow-up information were variable, potentially introducing information bias. Based on available data, no survival analysis was conducted since a substantial proportion of patients were lost to postoperative follow-up, with missing data at 6 and 12 months after surgery, mainly because adjuvant treatment was continued at other institutions. Additionally, several patients died within three months after surgery, and detailed data regarding the cause of death were unavailable. In addition, HER2 and HER3 immunohistochemical determinations are not included in standard diagnostic panels and were performed exclusively for research purposes. The higher HER2/HER3 positivity rates observed in our cohort (Table 1) compared to previous studies [15,31,39] may reflect methodological differences, including antibody selection, scoring criteria, and staining sensitivity, as well as differences in cohort composition and tumor sampling. The exclusive inclusion of treatment-naïve, resected PDACs from a high-volume referral center may also have contributed to this discrepancy. To address potential concerns regarding score aggregation, the primary objective of our analysis was descriptive and exploratory, aiming to assess overall receptor prevalence using a sensitive detection threshold, which may serve as the foundation for future research for establishing prognostic and predictive cut-offs. The cut-off values should be determined on a larger cohort to define clinically actionable scoring criteria in PDAC. Results reported in this study should be interpreted as hypothesis-generating and underscore the need for larger, prospectively designed studies to clarify the clinical relevance of HER2 and HER3 expression, to define standardized scoring criteria, and to explore their potential role in guiding personalized neo(adjuvant) therapeutic strategies in pancreatic cancer.

HER2 and HER3 are oncogenes that modulate cancer cell proliferation, invasion, and apoptosis. The function of pan-ErBB receptors as prognostic markers of pancreatic cancer remains a matter of active discussion. Our results highlight the need for further investigation of the therapeutic potential of these combinations in pancreatic cancer therapy, which will certainly include simultaneous targeting of many members of the HER family.

## 5. Conclusions

HER2 and HER3 overexpression in our PDAC cohort shows similar frequency among males and females. HER2+ was observed in 25 patients and was associated with 84% lymphovascular and perineural invasion. In HER3+ cases, venous tumoral thrombi were present in almost 40% of cases, and half of these patients had perineural invasion. Combined HER2 and HER3 positivity was identified in 19 cases (18%) and was frequently accompanied by perineural invasion (84.2%). This observation supports the involvement of the ErbB receptor family in pancreatic carcinogenesis and highlights its potential relevance as a therapeutic target, warranting further investigation.

## Figures and Tables

**Table 1:** Characteristics of the investigated cohort.

Characteristic	All (n = 106)	Male (n = 59)	Female (n = 47)	Stat. (*p*-Value)
Age, years ^a^	63 (9.6)	61.5 (10.1)	65 (8.6)	−1.9 (0.062)
Differentiation grade ^b^				4.4 (0.112)
G1	24 (22.6%)	16 (27.1%)	8 (17%)	
G2	65 (61.3%)	31 (52.5%)	34 (72.3%)	
G3	17 (16%)	12 (20.3%)	5 (10.6%)	
Tumor volume, cm^3 c^	6.6 [3.9 to 11.6]	6.5 [3.1 to 12.2]	6.8 [4.6 to 11]	−0.8 (0.440)
T stage ^b^				n.a. (0.621)
T1	9 (8.5%)	6 (10.2%)	3 (6.4%)	
T2	63 (59.4%)	36 (61%)	27 (57.4%)	
T3	33 (31.1%)	16 (27.1%)	17 (36.2%)	
T4	1 (0.9%)	1 (1.7%)	0 (0%)	
N ^b^				0.6 (0.748)
0	17 (16%)	10 (16.9%)	7 (14.9%)	
1	52 (49.1%)	27 (45.8%)	25 (53.2%)	
2	37 (34.9%)	22 (37.3%)	15 (31.9%)	
M ^b^				n.a. (0.042)
1	3 (2.8)	0 (0)	3 (6.4%)	
X	103 (97.2%)	59 (100%)	44 (93.6%)	
Lymphatic invasion ^b^	83 (78.3%)	48 (81.4%)	35 (74.5%)	0.7 (0.393)
Vascular invasion ^b^	47 (44.3%)	25 (42.4%)	22 (46.8%)	0.2 (0.648)
Perineural invasion ^b^	78 (73.6%)	42 (71.2%)	36 (76.6%)	0.4 (0.530)
Resection margins ^b^				0.1 (0.715)
R0	91 (85.8%)	50 (84.7%)	41 (87.2%)	
R1	15 (14.2%)	9 (15.3%)	6 (12.8%)	
Death ^b^	9 (8.5%)	6 (10.2%)	3 (6.4%)	n.a. (0.617)
HER2 positive ^b^	25 (23.6%)	14 (23.7%)	11 (23.4%)	0.002 (0.969)
HER3 positive ^b^	63 (59.4%)	34 (57.6%)	29 (61.7%)	0.2 (0.671)
HER2 or HER3 positive ^b^	69 (65.1%)	37 (62.7%)	32 (68.1%)	0.3 (0.564)
HER2 and HER3 positive ^b^	19 (17.9%)	11 (18.6%)	8 (17%)	0.05 (0.829)

Stat. represents the test statistics. ^a^ Data are expressed as mean (standard deviation) and comparison is made with the Student’s *t* test for independent samples; ^b^ data are reported as number (%), and comparison is performed with the Chi-squared test or Fisher’s exact test (n.a. denotes the absence of test statistics) according to the expected values; ^c^ data are reported as median [Q1 to Q3], where Q is the value of the quartile, and comparison between groups are made with the Mann–Whitney test; G1—well differentiated (low-grade malignancy), G2—moderately differentiated (intermediate grade), G3—poorly differentiated (high grade), G4—undifferentiated (high grade); TNM classification; resection margins: R0—free of tumor tissue or R1—with microscopic tumor tissue present; death—in-hospital death if it occurred due to cancer-related causes.

**Table 2:** Characteristics of the investigated cohort, stratified by HER2 status.

Characteristic	HER2 Positive (n = 25)	HER2 Negative (n = 81)	Stat. (*p*-Value)
Age, years ^a^	63.7 (9.7)	62.8 (9.6)	0.4 (0.674)
Differentiation grade ^b^			2.6 (0.274)
G1	8 (32%)	16 (19.8%)	
G2	15 (60%)	50 (61.7%)	
G3	2 (8%)	15 (18.5%)	
Tumor volume, cm^3 c^	6.5 [4.1 to 12.6]	6.6 [3.8 to 9.9]	0.5 (0.637)
T stage ^b^			n.a. (0.425)
T1	4 (16%)	5 (6.2%)	
T2	13 (52%)	50 (61.7%)	
T3	8 (32%)	25 (30.9%)	
T4	0 (0%)	1 (1.2%)	
N ^b^			n.a. (0.693)
0	3 (12%)	14 (17.3%)	
1	14 (56%)	38 (46.9%)	
2	8 (32%)	29 (35.8%)	
M ^b^			n.a. (0.779)
1	0 (0%)	3 (3.7%)	
X	25 (100%)	78 (96.3%)	
Lymphatic invasion ^b^	21 (84%)	62 (76.5%)	0.6 (0.429)
Vascular invasion ^b^	13 (52%)	34 (42%)	0.8 (0.378)
Perineural invasion ^b^	21 (84%)	57 (70.4%)	1.8 (0.177)
Resection margins ^b^			1.02 (0.313)
R0	23 (92%)	68 (84%)	
R1	2 (8%)	13 (16%)	

Stat. represents the test statistics. ^a^ Data are expressed as mean (standard deviation), and comparison is made with the Student’s *t* test for independent samples; ^b^ data are reported as number (%), and comparison is performed with the Chi-squared test or Fisher’s exact test (n.a. denotes the absence of test statistics) according to the expected values; ^c^ data are reported as median [Q1 to Q3], where Q is the value of quartile, and comparison between groups are made with the Mann–Whitney test; G1—well differentiated (low-grade malignancy), G2—moderately differentiated (intermediate grade), G3—poorly differentiated (high grade), G4—undifferentiated (high grade); TNM classification; resection margins: R0—free of tumor tissue or R1—with microscopic tumor tissue present; death—in-hospital death if it occurred due to cancer-related causes.

**Table 3:** Characteristics of the investigated cohort, stratified by HER3 status.

Characteristic	HER3 Positive (n = 63)	HER3 Negative (n = 43)	Stat. (*p*-Value)
Age, years ^a^	63.1 (9.8)	62.9 (9.3)	0.1 (0.896)
Differentiation grade ^b^			0.2 (0.890)
G1	14 (22.2%)	10 (23.3%)	
G2	38 (60.3%)	27 (62.8%)	
G3	11 (17.5%)	6 (14%)	
Tumor volume, cm^3 c^	6.8 [3.9 to 13.8]	6.5 [3.9 to 9.9]	0.7 (0.750)
T stage ^b^			
T1	5 (7.9%)	4 (9.3%)	
T2	37 (58.7%)	26 (60.5%)	
T3	20 (31.7%)	13 (30.2%)	
T4	1 (1.6%)	0 (0%)	
N ^b^			1.4 (0.489)
0	8 (12.7%)	9 (20.9%)	
1	33 (52.4%)	19 (44.2%)	
2	22 (34.9%)	15 (34.9%)	
M ^b^			0.9 (0.350)
1	1 (1.2%)	2 (8%)	
X	62 (76.5%)	41 (164%)	
Lymphatic invasion ^b^	52 (82.5%)	31 (72.1%)	1.6 (0.200)
Vascular invasion ^b^	24 (38.1%)	23 (53.5%)	2.5 (0.117)
Perineural invasion ^b^	50 (79.4%)	28 (65.1%)	2.7 (0.102)
Resection margins ^b^			0.02 (0.962)
R0	54 (85.7%)	37 (86%)	
R1	9 (14.3%)	6 (14%)	

Stat. represents the test statistics. ^a^ Data are expressed as mean (standard deviation) and comparison is made with the Student’s *t* test for independent samples; ^b^ data are reported as number (%) and comparison is done with the Chi-squared test or Fisher’s exact test (n.a. denotes the absence of test statistics) according to the expected values; ^c^ data are reported as median [Q1 to Q3], where Q is the value of the quartile, and comparison between groups is made with the Mann–Whitney test; G1—well differentiated (low-grade malignancy), G2—moderately differentiated (intermediate grade), G3—poorly differentiated (high grade), G4—undifferentiated (high grade); TNM classification; resection margins: R0—free of tumor tissue or R1—with microscopic tumor tissue present; death—in-hospital death if it occurred due to cancer-related causes.

**Table 4:** Characteristics of the investigated cohort, stratified by combined HER2 and HER3 status.

Characteristic	HER2 and HER3 Positive (n = 19)	HER2 and HER3 Negative (n = 87)	Stat. (*p*-Value)
Age, years ^a^	62.7 (9.9)	63.1 (9.6)	−0.2 (0.871)
Differentiation grade ^b^			n.a. (0.513)
G1	6 (31.6%)	18 (20.7%)	
G2	11 (57.9%)	54 (62.1%)	
G3	2 (10.5%)	15 (17.2%)	
Tumor volume, cm^3 c^	6.3 [4 to 14.5]	6.6 [3.9 to 10.8]	0.2 (0.866)
T stage ^b^			n.a. (0.385)
T1	3 (15.8%)	6 (6.9%)	
T2	9 (47.4%)	54 (62.1%)	
T3	7 (36.8%)	26 (29.9%)	
T4	0 (0%)	1 (1.1%)	
N ^b^			n.a. (0.271)
0	1 (5.3%)	16 (18.4%)	
1	12 (63.2%)	40 (46%)	
2	6 (31.6%)	31 (35.6%)	
M ^b^			n.a (0.725)
1	0 (0%)	3 (3.4%)	
X	19 (100%)	84 (96.6%)	
Lymphatic invasion ^b^	16 (84.2%)	67 (77%)	n.a (0.657)
Vascular invasion ^b^	9 (47.4%)	38 (43.7%)	0.09 (0.769)
Perineural invasion ^b^	16 (84.2%)	62 (71.3%)	1.8 (0.246)
Resection margins ^b^			3.3 (0.220)
R0	18 (94.7%)	73 (83.9%)	
R1	1 (5.3%)	14 (16.1%)	

Stat. represents the test statistics. ^a^ Data are expressed as mean (standard deviation), and comparison is made with Student’s *t* test for independent samples; ^b^ data are reported as number (%), and comparison is done with the Chi-squared test or Fisher’s exact test (n.a. denotes the absence of test statistics) according to the expected values; ^c^ data are reported as median [Q1 to Q3], where Q is the value of the quartile, and comparison between groups are made with the Mann–Whitney test; G1—well differentiated (low-grade malignancy), G2—moderately differentiated (intermediate grade), G3—poorly differentiated (high grade), G4—undifferentiated (high grade); TNM classification; resection margins: R0—free of tumor tissue or R1—with microscopic tumor tissue present; death—in-hospital death if it occurred due to cancer-related causes.

## Data Availability

The original contributions presented in this study are included in the article. Further inquiries can be directed to the corresponding author.
